# Impedance Characteristics of Microfluidic Channels and Integrated Coplanar Parallel Electrodes as Design Parameters for Whole-Channel Analysis in Organ-on-Chip Micro-Systems

**DOI:** 10.3390/bios14080374

**Published:** 2024-08-01

**Authors:** Crystal E. Rapier, Srikanth Jagadeesan, Gad D. Vatine, Hadar Ben-Yoav

**Affiliations:** 1 Nanobioelectronics Laboratory (NBEL), Department of Biomedical Engineering, Faculty of Engineering Sciences, Ilse Katz Institute for Nanoscale Science and Technology, Zelman Center for Brain Science Research, Ben-Gurion University of the Negev, Building 64, Rm 204, Beer Sheva 8410501, Israel; rapier@post.bgu.ac.il; 2 Department of Physiology and Cell Biology, Faculty of Health Sciences, Regenerative Medicine and Stem Cell (RMSC) Research Center, Zelman Center for Brain Science Research, Ben-Gurion University of the Negev, Building 42, Rm 326, Beer Sheva 8410501, Israel; srikanth@post.bgu.ac.il (S.J.); vatineg@bgu.ac.il (G.D.V.)

**Keywords:** microfluidics, impedance-based sensor, design parameters, organ-on-a-chip, electric cell substrate impedance spectroscopy (ECIS)

## Abstract

Microfluidics have revolutionized cell culture by allowing for precise physical and chemical environmental control. Coupled with electrodes, microfluidic cell culture can be activated or have its changes sensed in real-time. We used our previously developed reliable and stable microfluidic device for cell growth and monitoring to design, fabricate, and characterize a whole-channel impedance-based sensor and used it to systematically assess the electrical and electrochemical influences of microfluidic channel boundaries coupled with varying electrode sizes, distances, coatings, and cell coverage. Our investigation includes both theoretical and experimental approaches to investigate how design parameters and insulating boundary conditions change impedance characteristics. We examined the system with various solutions using a frequency range of 0.5 Hz to 1 MHz and a modulation voltage of 50 mV. The results show that impedance is directly proportional to electrode distance and inversely proportional to electrode coating, area, and channel size. We also demonstrate that electrode spacing is a dominant factor contributing to impedance. In the end, we summarize all the relationships found and comment on the appropriateness of using this system to investigate barrier cells in blood vessel models and organ-on-a-chip devices. This fundamental study can help in the careful design of microfluidic culture constructs and models that require channel geometries and impedance-based biosensing.

## 1. Introduction

The field of microfluidics has revolutionized both mammalian and non-mammalian cell culture. Thanks to microfluidic devices, the physical and chemical environment of cells can be customized and studied, made more precise, and modeled after living systems, such as single nucleotide polymorphism detection [1], device material (paper) investigation [2], and nucleic acid amplification in droplets [3]. Microfluidic cell culture can control the flow of fluids [4,5,6], permit multiplexing [7], control the timing of reagents [7], oxygen saturation [8], and cause cellular polarization and transformation [9,10]. The level of microfluidic device functionality can be greatly increased when it is coupled with detection or sensing platforms for real-time analysis. Many sensing and detection platforms utilize electrochemical impedance spectroscopy (EIS) as their sensing technology [11,12,13,14]. EIS can determine the activities and responses of cells and tissues in a non-invasive dmanner using different electrical frequencies. EIS does so by measuring the impedance, or complex resistance, of a current by an electrode–electrolyte or electrode–cell interface. Impedance can be evaluated against frequency. It can respond differently depending on the conductive properties of the material, cell, tissue, or solution being measured, as well as the boundary and electrode conditions.

Examples of microfluidic cell culture devices that utilize integrated impedance sensors include electric cell-substrate impedance sensing (ECIS) and organ-on-a-chip (OOC) technologies. ECIS is an impedance-based monitoring technology that was developed by Keese and Giaever in 1991 [15,16,17]. ECIS involves the impedance monitoring of cells typically cultured on small thin film electrodes within polystyrene wells. The second example of microfluidic devices that have benefitted from impedance-based sensing approaches are OOCs. Organ-on-a-chip technology was first developed by Ingber and his team at the Wyss Institute [18,19,20,21,22]. OOCs are engineered constructs that mimic human tissue and organ function. These constructs help scientists understand phenomena such as basic cellular functions within a tissue; disease pathology; tissue regeneration; and drug pharmacokinetics. A special group of OOCs that mimic barrier interfaces, such as blood vessels (vessel-on-a-chip, VOC) or the blood–brain barrier (blood-brain-barrier-on-a-chip, BBBOCs), require microfluidic impedance platforms to probe the integrity of barrier-producing cells. VOCs and BBBOCs are typically configured by growing barrier-producing endothelial cells within microfluidic channels [22,23,24,25,26,27,28]. These cells form intercellular connections (tight junctions) between adjacent cells to produce a highly selective semipermeable barrier. Determining the level of barrier integrity is a critical step in the validation of these in vitro models. Impedance technology provides a means to quantitatively assess barrier integrity as well as monitor barrier (e.g., cell–cell, tissue–tissue, or tissue–blood) interactions for these model systems.

Solution resistance is used as a sensing parameter for many impedance-based sensors. Both solution resistance and impedance can be altered by device boundary conditions and electrode geometry because these variables can physically alter the current flow. Although impedance-based biosensing is a useful and powerful tool for microfluidic cell culture and OOCs, some fundamental questions remain. For example, how do bounded microfluidic channel dimensions. along with other device design parameters, affect impedance and solution resistance? The relationships of channel width, electrode area, distance, and coatings to impedance need to be understood to make the sensing capabilities within microfluidic culture more effective and efficient. Knowing the effects that design parameters have on the impedance of a microfluidic system allows designers to anticipate and avoid problems, as well as intentionally influence cell culture. Therefore, answering the posed question is important because it can aid in the effective and intelligent design of future microfluidic engineered constructs and cell culture devices. This is especially true for BBBOC, VOC, or any other OOC model system that relies on impedance-based sensing for quantitative barrier measurements and real-time monitoring within microfluidic channels.

We previously designed a microfluidic system that was capable of whole-channel impedance analysis using integrated linear coplanar gold electrodes [29]. Our objective here is to examine design rule relationships for impedance results and to also provide a full system characterization with various microfluidic channel sizes. Our investigation includes both theoretical and experimental approaches to answer the design parameter versus impedance question. This microfluidic system, with the exclusion of cells, serves as an electrolyte conductivity sensor. As impedance and solution resistance are sensitive to geometry, we leveraged the use of a conductivity sensor to examine the effect of different microfluidic design parameters. We report findings on the influences of microfluidic channel dimensions, electrode spacing, size, and electrode coatings (matrix and cells) on AC impedance responses with this type of electrode setup. The data from our investigation are intended to provide basic design guidelines and relationships with biological cell material to decrease the impedance baseline and maximize the biological information for similar geometries. In addition, the device design is intended to be simple in its creation for others to use directly or to integrate its parameters into their microfluidic cell culture systems.

## 2. Materials and Methods

### 2.1. Fabrication of Microfluidic Devices with Integrated Sensors

#### 2.1.1. Silicon Mold Fabrication for Microfluidic Channels

Microfluidic channels were fabricated using standard photolithography techniques. The photolithography technique was chosen in this study to provide high-resolution control for the geometric properties of the device as opposed to low-resolution techniques, such as 3D printing. Channels were first designed using CleWin CAD software (version 4.0; WieWeb software, Hengelo, The Netherlands) and printed as a photomask through Artnet Pro, Inc. (San Jose, CA, USA). To make channel features into a mold, a four-inch silicon wafer was spin-coated with SU-8 3050 photoresist (Kayaku Advanced Materials, Westborough, MA, USA) and soft-baked for 15 min at 95 °C. The spin-coated wafer was then covered with the photomask containing the microfluidic channel designs and then exposed to UV light at 250 mJ/cm^2^. The UV light reacts with the photoresist, resulting in the transfer of the photomask image. The silicon wafer was subsequently baked post-exposure, immersed in an AZ EBR developer (Merck Performance Materials GmbH, Wiesbaden, Germany), and cleaned with isopropanol. The steps are in accordance with Kayaku Advanced Materials, Inc. processing guidelines to achieve 55 µm high channel features.

#### 2.1.2. Electrode Fabrication

Electrode photomasks were also designed on CleWin4 software and printed through CAD/Art Services, Inc. (Bandon, OR, USA). A photolithography lift-off technique and E-beam evaporation were utilized to make thin film electrodes on glass substrates. Briefly, a four-inch borosilicate glass wafer was spin-coated with the image-reversal photoresist AZ-5214E. The borosilicate glass wafer was then soft-baked, briefly exposed to UV light, baked post-exposure, UV-flood-exposed, and finally immersed in an MIF726 developer to bring out the electrode design features according to Merck’s technical guidelines through Kayaku Advanced Materials, Inc. (Westborough, MA, USA). The glass wafer was then deposited with 20 nm of titanium, followed by a 200 nm layer of gold. After metal deposition, glass wafers were subsequently soaked in acetone for an hour to complete the lift-off process. The glass wafers with electrodes were diced into individual pieces, or “chips”, and stored for further device fabrication.

#### 2.1.3. Microfluidic Device Assembly and Preparation

We used our previously developed method to manufacture 3 microfluidic devices in a reproducible and reliable manner and to grow cells in the channels in a confluent conformation [29]. Microfluidic channels were made using the polydimethylsiloxane (pdms) replica molding process with the silicon molds described above. A 10:1 pdms base to curing agent ratio was mixed thoroughly, poured onto silicon molds, and degassed in a vacuum chamber. The silicon molds were then placed into an 80 °C oven for a minimum of two hours. Once the pdms solidified, microfluidic channels were cut and removed from the mold using a scalpel blade. Inlet and outlet holes were made into the pdms channels with a 2 mm biopsy punch. To assemble the complete microfluidic system, pdms channels and electrode chips were plasma-treated, aligned, and placed into contact with each other. For devices testing the impedance relationships for the electrode coating, microfluidic channels were first coated with the Emulate^®^ reagent, cured, coated with a 4:1 mixture of 1 mg/mL human placenta type IV collagen (Sigma-Aldrich, Darmstadt, Germany) to 1 mg/mL fibronectin (Sigma-Aldrich, Darmstadt, Germany), and placed into a 37 °C incubator for 12 h. This procedure was also followed prior to cell-seeding experiments in addition to device sterilization with UV light. Emulate^®^ reagent assists in the binding of the extracellular matrix (ECM) to glass substrates. Each microfluidic device was freshly fabricated prior to the experiment and showed reliable characteristics with no aging.

### 2.2. Experimental Setup and Electrochemical Impedance Spectroscopy

Fabricated microfluidic devices were filled with various solution samples and placed inside a Faraday cage. Potentio electrochemical impedance spectroscopy was performed with a VSP-300 potentiostat (Biologic Scientific Instruments, Seyssinet-Pariset, France). EC-Lab software v11.32 provided by Biologic Scientific Instruments was used to analyze impedance spectra. Fitting was also performed using this commercial EC-Lab software. Impedance measurements were conducted with frequencies between 0.5 HZ and 1 MHz. The modulation (AC) voltage was set to 50 mV. Impedance measurements of cell media were taken at 37 °C. These conditions were defined based on our previous research [29].

### 2.3. Cell Culture

Induced pluripotent stem-cell-derived brain microvascular endothelial-like cells (iBMECs) were tested as part of the design parameters. iBMECs were reprogrammed from human omental stromal cells (a gift of Dr. Rivka Ofir of Ben Gurion University, Beer Sheva, Israel). The differentiation of reprogrammed cells required a two-to-three-day passage over Matrigel in NutriStem™ medium until 25–30% confluency was achieved. Six days following this, an unconditioned medium lacking the basic fibroblast growth factor (bFGF, PeproTech, Rehovot, Israel) was used as the cell medium. After two days of growth, a human endothelial serum-free medium (hESFM; Life Technologies Corporation, Grand Island, NY, USA) with 20 ng/mL of bFGF and 10 mM of all-trans retinoic acid (RA, Sigma, Darmstadt, Germany) was added. The cells were then gently dissociated and incubated with Accutase for up to 35 min before seeding into microfluidic devices. iBMECs were subsequently grown in an endothelial cell medium without bFGF and RA. These conditions were defined based on our previous research [29].

## 3. Results and Discussion

### 3.1. The Device Design

**Figure 1:**
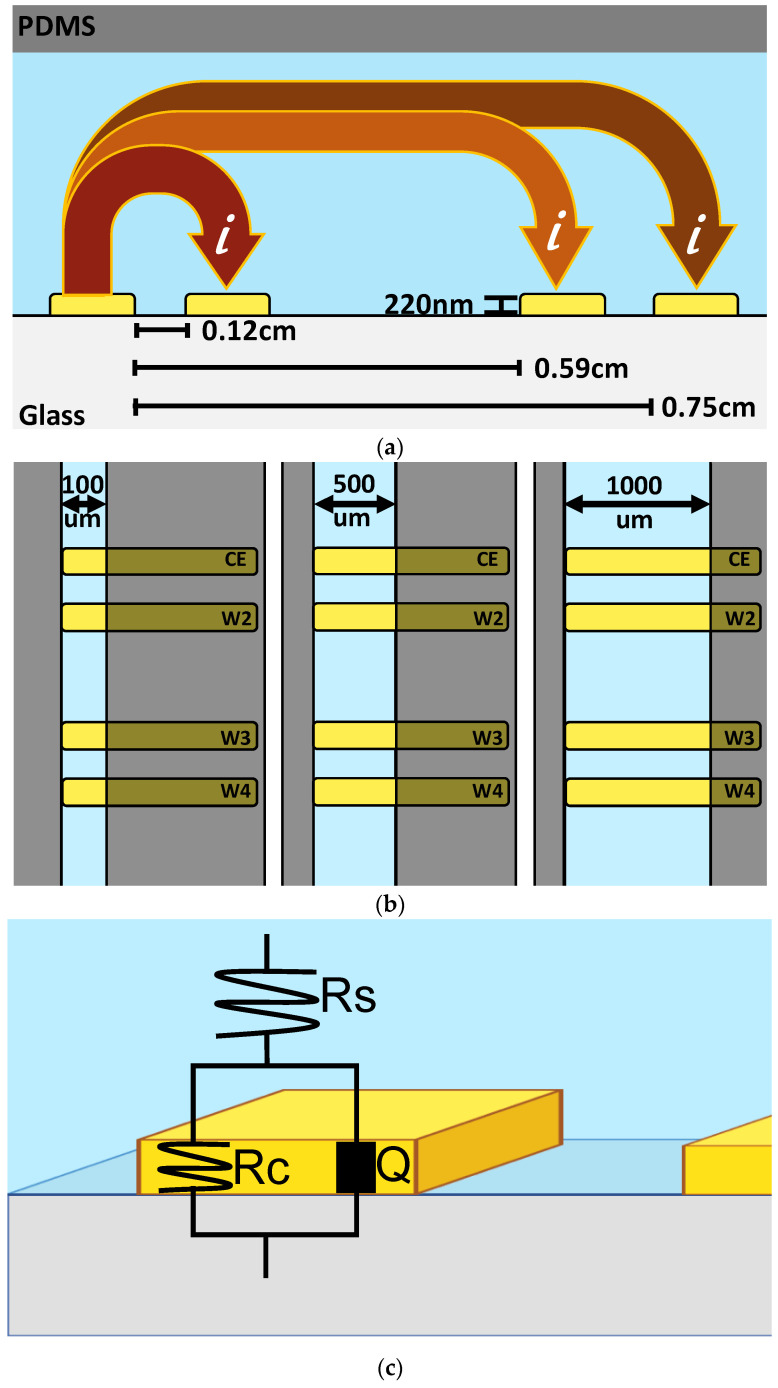
Schematics of electrode and microfluidic channel layout. (**a**) Cross-section of the device showing current flow, electrode thickness, and distance. (**b**) Microfluidic channel widths and electrode layout with CE as the counter electrode and W2–W4 as the working electrodes. (**c**) Schematic representing a circuit model for an electrode–electrolyte interface.

Microfluidic devices with various channel sizes (55 µm height, 100–1000 µm wide) covering integrated linear coplanar thin-film gold electrodes were successfully created. This design was chosen due to its popular use in organ-on-chip micro-systems and its provision of the spatiotemporal resolution of cell growth [29]. Moreover, the device was reproducibly fabricated and showed stable performance under the introduction of various solutions for several days. In total, 400 µm wide gold electrode bands were designed to span the entire width of each channel size. A linear coplanar parallel electrode design should produce a uniform current distribution along the length of microfluidic channels. This is necessary for a robust fundamental investigation of changes in impedance and solution resistance. The parallel electrode design was chosen to cover a greater surface area. Various combinations of paired electrodes can be activated throughout the length of the microfluidic channel. The spacing of the electrodes allows the sensing of a wide or narrow range of length. Figure 1a,b is a demonstration of the system’s channels, electrode design parameters and impedance workflow with the current (i) flowing from the counter electrode (CE) to the selected working electrode (WE). An electrode combination termed “W2” represents electrode pair one and two, with electrode one serving as the CE. Combinations W3 and W4 represent electrode pairs one and three and one and four, respectively. Electrode pair W4 covers a range of 0.8 cm, which is the furthest distance measured for impedance measurements within the microfluidic system described here.

### 3.2. Theoretical Approach

#### 3.2.1. Equivalent Circuit Modeling

Equivalent circuit modeling was utilized to obtain parameters such as solution resistance (Rs), charge transfer resistance, etc. The microfluidic sensing system described herein can be considered an electrochemical cell. Therefore, the system’s components can be represented as a combination of elements (resistors, capacitors, etc.) in an electrical circuit model, each representing a physical process. The quantitative value of each circuit element (system component under evaluation) can be obtained through circuit models. Basic electrode systems can be represented with a Randles circuit, which is an empirical model that describes a simplistic electrochemical interface. Generally, it is best to start with a simple representation and then build to a more complex model of the system under investigation. The ideal circuit elements can be changed depending on the system’s details. This is necessary when these ideal basic elements do not fully reflect system components. For example, we used a constant-phase element (CPE) versus a capacitor to represent the double layer of charge generated on an electrode surface. The CPE allowed for the accommodation of system fluctuations (electrode topography, for example), nonlinearities, the dependence of system elements on frequency, and other irregularities that could not be matched to ideal elements. The substitution of a capacitor element for a CPE also improved the model’s fit to experimental data. Figure 1c is a schematic representing one of the circuit models used in our investigation. The electrode–electrolyte interface is modeled by setting the CPE in parallel to a charge-transfer resistance (Rc) element, both of which are in series to a solution resistance (R) element.

#### 3.2.2. Derivation

Derivations of Ohm’s Law are used to examine the theoretical relationships between resistance, current, voltage, channel dimension, and the electrode area. In Ohm’s Law, the current, *i*, resulting from a given AC voltage, *v*, when applied to electrodes, can give rise to the value of electrical resistance in Equation (1). When measuring dielectric material, Ohm’s Law can be represented as solution resistivity, *ρ* [Ωcm], the length of the dielectric material, *L* [cm], and the area of two equivalent electric contacts, *A* [cm^2^].

(1) $$R=\frac{v}{i}=\frac{\rho L}{A}$$

This equation can also be applied to two coplanar electrodes of equal surface area, *S*. The electrolyte resistance (*R_solution_*) is given by the relationship in Equation (2), where an electrolyte of a certain resistivity, *ρ*, is bound between two electrodes of equal area, *S*, with a distance, *D*, between them.

(2) $$R_{solution}=\frac{D\;\rho }{S}$$

Applying Equation (2) to a microfluidic setup containing a channel covering a pair of parallel coplanar electrodes spanning the width of a channel gives rise to Equation (3). In this equation, the resistance of the solution within the microfluidic channel is related to the channel dimensions, where *D* becomes *l*, the length of the channel, and is divided by the channel’s cross-sectional area (*w·h*), which substitutes *S* in Equation (2).

(3) $$R_{channel}=\frac{l\;\rho }{wh}$$

To compare the effects of channel dimensions such as width, *l*, *h*, and *ρ* can be pulled out into a single constant, *n*. This makes the solution resistance within the channel inversely proportional to the channel width (Equation (4)).

(4) $$R_{channel}=\frac{n}{w}$$

For example, if *w* were to increase in Equation (4), the current conduction would increase, and the resistance through the microfluidic channel could theoretically decrease.

### 3.3. EIS, Electrode Characterization, and Electrolyte Interface Analysis

The microfluidic system’s electrode functionality and electrode–electrolyte solution interface characteristics were examined and validated by testing four control solutions: deionized water (dH20), ferricyanide/ferrocyanide (FeFe), phosphate-buffered saline (PBS), and cell media. Deionized water was used to test the electrode performance in the presence of a weak electrolyte. Ferricyanide/ferrocyanide, [Fe(CN)_6_]^3−^/[Fe(CN)_6_]^4−^, is a standard electroactive redox probe. This redox couple is used to evaluate the electron transfer capabilities of electrodes as well as monitor the stepwise modification of electrode surfaces. PBS is commonly used in biological culture to test biosensors. The isotonic ion concentration and osmolarity of PBS are formulated to match human body fluids such as blood plasma. Cell media were tested to examine their conductive properties and suitability for EIS; because it is required for cell culture experiments, determining the impedance characteristics of cell media is an informative control. The examination of cell media was conducted at 37 °C because it is a condition needed for cell culture experiments and because temperature is known to influence resistance. Other control experiments were carried out in ambient conditions. All experiments were performed within a Faraday cage to mitigate interference with the impedance measurements. Impedance measurements were taken between different pairs of gold electrodes along the length of the microfluidic channel. Impedance analysis and fitting were accomplished using the commercial EC-Lab software v11.32.

**Figure 2:**
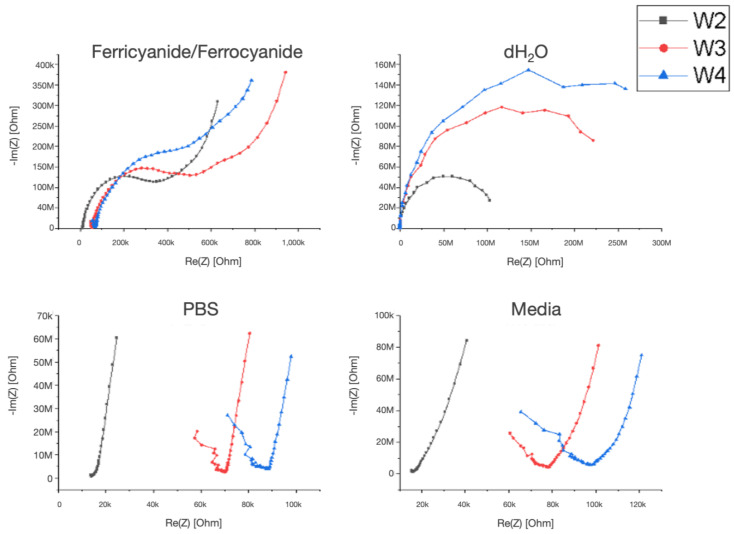
Nyquist plots characterizing the electrode–solution interface and the effects of interelectrode spacing on the system. W2, W3, and W4 correspond to 0.12 cm, 0.59 cm, and 0.75 cm electrode spacing, respectively.

The impedance results characterizing electrode functionality with control solutions (controlled pH and ionic strength in a human endothelial serum-free medium) are presented in Figure 2. Electrodes functioned properly for all the solutions tested. The results are from a 1000 µm wide microfluidic channel device. Ion travel distance was observed by changing the interelectrode spacing. W2–W4, which corresponds to an increasing electrode pair distance. W2 corresponds to electrode pair one and two, which are 1200 µm apart. W3 corresponds to electrodes one and three with a 5900 µm distance apart, and W4 represents electrodes one and four with a 7500 µm distance. Impedance increases as the distance between the working and counter electrode increases. This was found for all solutions tested. The Nyquist plots in Figure 2 represent typical plot trends, as seen for all the channel sizes tested.

### 3.4. Electrical Responses and Relationships between Channel Dimension, Electrode Area, Interelectrode Spacing, and Impedance

**Figure 3:**
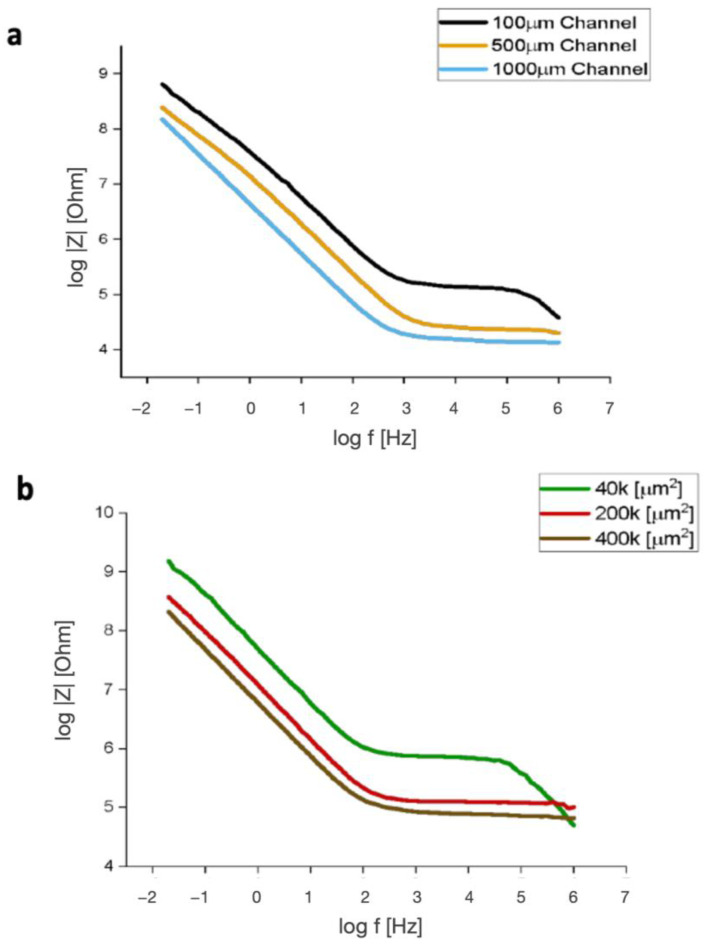
Bode plots of total impedance against frequency. (**a**) Plot comparing frequency characteristics of different channel widths. (**b**) Plot comparing frequency characteristics of different electrode areas.

The frequency response of microfluidic systems with different channel widths and electrode areas was examined and is presented in Figure 3 as Bode plots. The frequency characteristics were tested using PBS (Figure 3a) or cell media (Figure 3b) on bare gold electrodes. Changes in channel widths and electrode area are clearly differentiated in both the high and the low-frequency regions of the Bode plots. As a channel’s width increased, the system’s total impedance decreased in magnitude. This is also true for the electrode geometric area. A horizontal line, indicative of resistive behavior, is seen starting at log 3 Hz for the channel width Bode plot and at log 2 Hz for the electrode area Bode plot. The system’s resistive behavior starts at a lower frequency for the channel width plot compared to the electrode area plot. A negative slope is seen below log 2 Hz and log 3 Hz for each Bode plot in Figure 3 and is indicative of capacitive behavior within the system.

**Figure 4:**
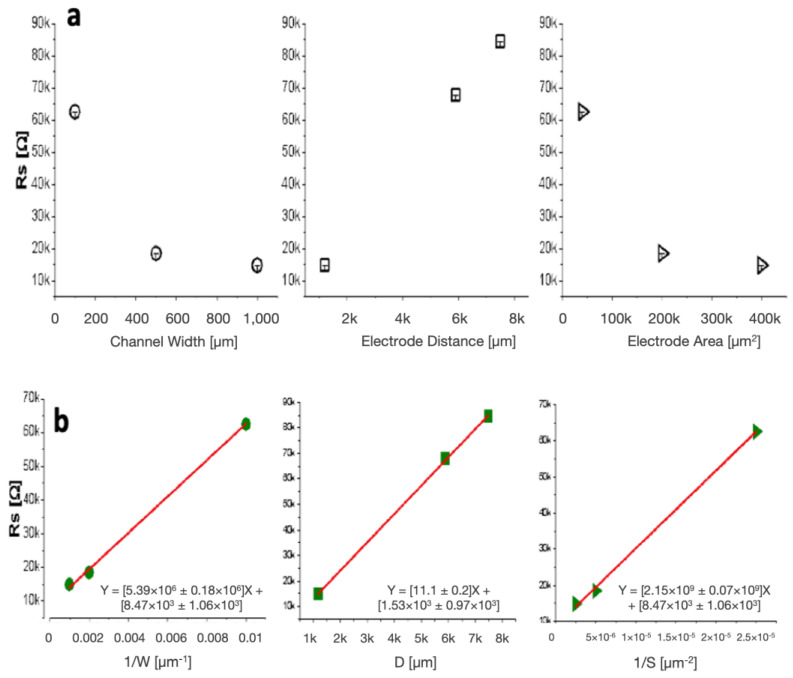
Relationships of channel and electrode parameters on solution resistance. (**a**) Solution resistance versus changes in channel width (**left**), electrode distance (**middle**), and electrode area (**right**). (**b**) Linear relationships of solution resistance to channel width (**left**), electrode distance (**middle**), and area (**right**).

We next examined the effect of varying interelectrode spacing (electrode distance), electrode area, and channel widths on solution resistance using PBS as the model solution. The microfluidic channels tested had the same heights but varying widths. An inverse relationship was also found between solution resistance and channel width (Figure 4a, left). The linear regression of the results shows how the relationship follows the expected proportionality in Equation (4), validating experimental data (Figure 4b, left).

The effect of electrode distance was examined by measuring various distances between electrode pairs within 1000 μm wide microfluidic devices. The electrodes within the channel had the same area. We found that as the distance between electrodes increased, the solution resistance increased in a linear fashion (Figure 4a, middle). This linear relationship is also seen in Figure 4b (middle) and correlates with the direct proportionality between solution resistance and the distance between electrodes theorized in Equation (2).

A negative slope was found when comparing solution resistance to the varying electrode area (Figure 4a, right). This follows the expected inversely proportional relationship in Equation (1) and is verified with linear regression (Figure 4b, right). As the electrode area increased, the solution resistance decreased. This is because there is an increase in the effective area of the electrolyte, allowing for the avoidance of charge collection at the electrode surface. The distance between electrodes remained constant for each device tested.

### 3.5. Electrical Responses of Coplanar Electrode Coverage

**Figure 5:**
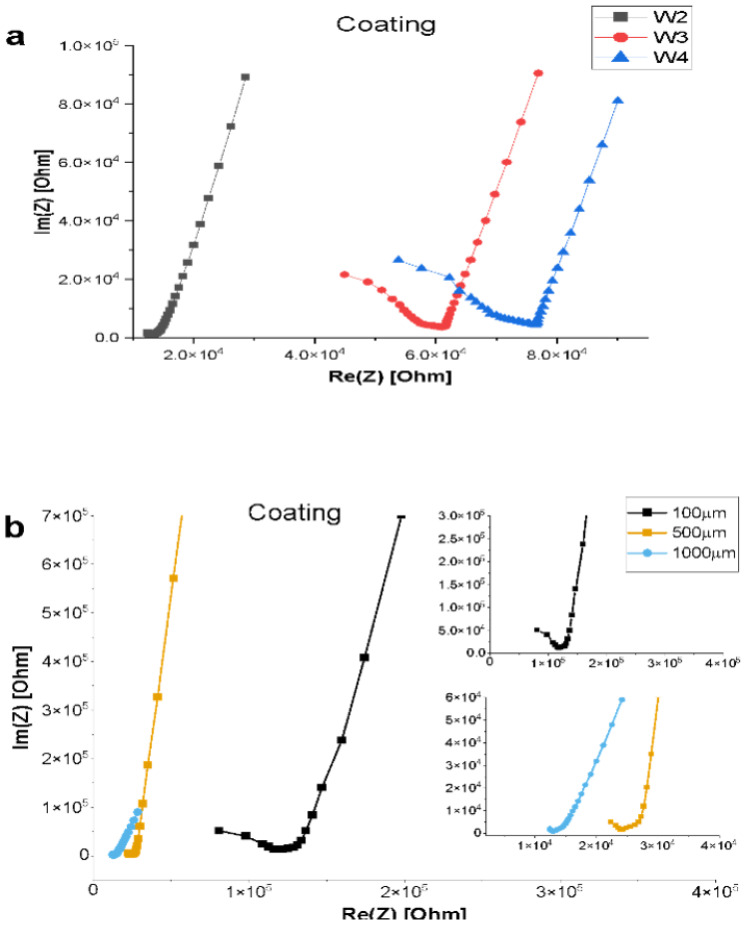
Effect of ECM electrode coating on impedance. (**a**) Impedance response to electrode distance. (**b**) Impedance response to changes in channel width. Insets show low- and high-frequency regions of the Nyquist plot.

The effect of the electrode coating on the electrical properties of microfluidic devices was also tested. Electrodes were coated with an extracellular matrix (ECM), a common 3D network used for cell-substrate attachment in many cell culture experiments. The Nyquist plot in Figure 5a shows ECM-coated electrodes following the same trend as bare electrodes in terms of increasing impedance as the electrode distance increases. An inversely proportional relationship was observed between impedance and ECM-coated electrodes of the same spacing within different channel widths (Figure 5b). The Nyquist plot in Figure 5b also reveals the 100 μm channel device to have a tenfold higher impedance than 1000 μm devices.

**Figure 6:**
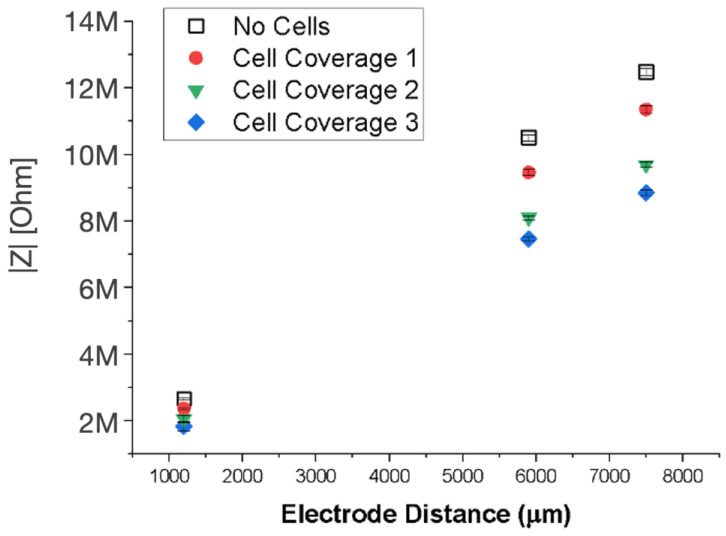
Impedance versus electrode distance for ECM-coated electrode devices with and without cells. “Cell coverage 1”, “2”, and “3” are devices containing an increasing number of cells, respectively.

Cell coverage was also examined as part of the investigation into the microfluidic system’s design parameters. The effects that cell coverage and interelectrode spacing have on impedance are presented in Figure 6. A positive correlation between increasing electrode spacing and impedance was found for both ECM-coated devices with and without cells. However, as ECM-coated electrodes were covered with more cells (cell coverage 1–3 increasing, respectively), a decrease in impedance was observed. It is evident from the data in Figure 6 that electrode distance is the dominating factor contributing to an increase in impedance. Therefore, it would be necessary to only make comparisons of impedance and cell number between the same rather than different electrode pair distances.

### 3.6. Summary of Design Rule Relationships and Future Outlook

Microfluidic system parameters and electrical relationships in terms of impedance and solution resistance are summarized in Table 1. Total impedance increases inversely with electrode ECM coating, cell coverage, electrode area, and microfluidic channel width. However, total impedance is directly proportional to changes in electrode distance. Solution resistance is also directly proportional to changes in electrode distance, but it is inversely proportional to changes in channel width and electrode area. Many sensors use solution resistance as a sensing mechanism. Without cells, our microfluidic system performs as an electrolyte conductivity sensor and allows for an investigation into the electrical influences of electrodes and microfluidic channels. With cells, however, the system’s integrated coplanar parallel electrode layout can be used to acquire impedance and other electrical information from a culture of adherent cells grown along its channel. VOCs and BBBOCs are fabricated with endothelial cells housed within microfluidic channels. These cells are tested for their barrier strength with the use of impedimetric techniques. Our reported design rules are pertinent to BBBOC and VOCs that rely on microfluidic impedance-based sensing capabilities to measure and analyze the integrity of barrier-producing cells grown within microfluidic channels. The coplanar design rules herein can provide a baseline with which to compare and normalize microfluidic channel cell culture data. Our design rule findings can also serve as a foundation for predictive microfluidic impedimetric sensor behavior, which is useful for the intentional and directed control of impedance response.

## 4. Conclusions

Microfluidic devices with integrated linear coplanar gold electrodes were fabricated for whole-channel impedance analysis and used to systematically investigate how microfluidic channel boundaries and integrated electrode design affect impedance-based sensing. The multiple parallel coplanar design allows for the flexible assessment of the entire microfluidic channel. EIS combined with equivalent circuit analysis and derivations of Ohm’s Law for electrical relationships were utilized to obtain the results. Our results show that impedance and other electrochemical parameters are proportionally influenced by the microfluidic channel and integrated electrode designs. We also observed how coplanar electrode spacing is a dominant factor contributing to an increase in impedance, which is the inverse of increasing cell coverage. This work contributes to the fundamental understanding and technical basis of microfluidic channels and OOC design rules. We hope the design rules will help with the intelligent design and increased efficacy of microfluidic in vitro models with sensing capabilities. A fundamental understanding of the effects that channel dimension and electrode design have on impedance can also help drive precise microfluidic culture design. In the end, we summarized all the relationships found and commented on the appropriateness of using this system to measure impedance within the microfluidic-engineered vessel and BBBOC devices. Investigating other design parameters, such as the biochemical properties of the solution (e.g., pH and ionic strength) and the electrode material (e.g., carbon electrodes and charged film modifications), will enable the design of micro-devices with high and controlled performance for cell growth and monitoring.

## Figures and Tables

**Table 1:** Design rule relationships for coplanar parallel electrodes in microfluidic channels.

Z	↑	As Electrode Distance ⇧
Z	↑	As channel width ⇩
Z	↓	With electrode coating
Z	↓	As electrode area ⇧
Z	↓	With ⇧ in cells between electrodes
R_s_	↑	As electrode distance ⇧
R_s_	↓	As channel width ⇧
R_s_	↓	As electrode area ⇧

## Data Availability

Data will be provided upon request from the corresponding author.
